# Looking ahead in retinal disease management: *highlights of the 2019 angiogenesis, exudation and degeneration symposium*

**DOI:** 10.1186/s40942-019-0174-y

**Published:** 2019-05-29

**Authors:** Carmen A. Puliafito, Charles C. Wykoff

**Affiliations:** 1 Los Angeles, USA; 2 Houston Methodist Hospital and Weill Cornell Medical School, Houston, USA

The 15th annual Angiogenesis, Exudation and Degeneration Symposium sponsored by the Bascom Palmer Eye Institute, Miami, Florida, was held on February 9th, 2019. This program is a leading international forum for the presentation of new therapies for age-related macular degeneration (AMD) and diabetic retinopathy (DR) which together account for more than 80% of cases of bilateral blindness in the industrialized world [1]. Lengthening lifespans coupled with an increasing global prevalence of diabetes translates [2] into projections of increasing societal burdens related to these diseases over the next two decades and beyond.

Widespread use of anti-VEGF pharmacotherapies has dramatically improved the prognosis for patients with the neovascular form of AMD (nAMD) [3] and DR [4]. Intravitreal injections of anti-VEGF agents are now the most commonly performed ophthalmic procedure. We describe 5 key themes and highlight specific topics of interest to the vitreoretinal community within each of these themes.

## Reducing the anti-VEGF treatment burden

Reducing the treatment burden associated with regular anti-VEGF intravitreal injections is a priority. nAMD and DR are chronic, relapsing disorders [5, 6]. Patients may require tens of injections over many years of treatment. Compliance with such a demanding regimen is challenging. Current promising approaches include (a) new hardware to deliver anti-VEGF medications (b) new pharmaceuticals with longer durability of biological effect (c) novel formulations of anti-VEFG agents for sustained release and (d) gene therapy.

Dr. Carl Regillo presented the LADDER phase II trial results studying the port delivery system (PDS, Genentech) [7]. The PDS is surgically implanted at the pars plana. When it is filled with ranibizumab, it is reported to be able to provide continuous delivery of the drug by passive diffusion with external refills delivered in the clinic through the overlying conjunctiva and Tenon’s layer. The phase II outcomes appeared positive. In the 100 mg/ml dose arm: (a) 80% of patients went 6 months or longer before first refill of the device, (b) the median time to first refill was 15 months, and (c) BCVA and anatomic outcomes were comparable to those achieved with monthly intravitreal ranibizumab. A Phase III trial program is underway [8].

Dr. Pravin Dugel reported the results of a post hoc study of macular drying achieved with aflibercept compared to brolucizumab in the HAWK and HARRIER phase III randomized clinical trials (Novartis) [9]. While VA outcomes throughout the trial were essentially identical with the different anti-VEGF agents, brolucizumab achieved superior reductions in central subfield thickness (CST) from baseline to Week 16 and Week 48, and this difference was maintained at Week 96: fewer patients treated with brolucizumab had intraretinal and/or subretinal fluid. The proportion of eyes that remained fluid free for three or more visits after becoming fluid free was higher in the 6 mg brolucizumab group (54.5%) compared to the 2 mg aflibercept group (42.7%). The clinical significance of this enhanced drying of the central macula is under discussion.

Dr. David Boyer presented phase I/IIa trial results of sunitinib (GB-102) (Graybug Vision) [10]. Sunitinib is a multi-targeted, receptor tyrosine kinase inhibitor which reportedly inhibits all VEGF receptor types. The drug is formulated in injectable microparticles, which aggregate inside the vitreous cavity following intravitreal injection into a depot which subsequently slowly, releases active drug over months. This study enrolled previously anti-VEGF treated nAMD eyes with persistent disease activity (intraretinal or subretinal fluid), which were treated with a single dose of GB-102 and followed for 8 months. In the study, 46% of eyes at 8 months demonstrated sustained treatment effect following GB-102 treatment, meaning that they did not receive anti-VEGF rescue therapy. Migration of microparticles into the anterior chamber were observed in 9 eyes (28%) and was managed by observation or anterior chamber washout. The upcoming phase IIb trial will use microparticles manufactured differently in attempt to decrease the possibility of anterior particle migration.

Two presentations described data related to gene therapy approaches to nAMD. Dr Peter Campochiaro described an approach using an adeno-associated virus (AAV)-8 vector delivered by sub-retinal injection during a pars plana vitrectomy carrying a gene encoding a molecule similar to ranibizumab, an anti-VEGF fragment antigen-binding (Regenxbio) [11]. Dr David Brown described an approach using an AAV.7mB vector delivered by intravitreal injection carrying a gene encoding a molecule similar to aflibercept (Adverum) [12]. Both approaches have shown promising results with reliable gene expression and protein production in animal models and early phase human studies are underway.

## Prevention of progression to advanced disease states

Prevention of progression of DR and intermediate AMD has been explored with mixed results. In DR, chronic anti-VEGF therapy can improve retinopathy severity scores and reduce disease progression. How to apply these findings in real-world clinical situations is under discussion. In intermediate AMD, anti-VEGF therapy in high-risk dry AMD eyes does not appear to decrease the rate of conversion from the intermediate stage to nAMD.

Dr. Charles Wykoff presented 1-year results from the phase III, PANORAMA trial that studied the use of aflibercept for the treatment of moderately severe to severe non-proliferative DR without center-involved diabetic macular edema (CI-DME) [13]. The study demonstrated an impressive regression in diabetic retinopathy severity and a reduction in the development of proliferative diabetic retinopathy (PDR) and CI-DME. Studied eyes (*n* = 402) had an average baseline VA of approximately 20/25, were anti-VEGF treatment naïve, and were randomized equally into three treatment arms: sham, aflibercept every 2 months following 5 monthly loading doses or aflibercept every 4 months following 4 loading doses. At 1 year, 80% of eyes in the every 2 months group and 65% of eyes in the every 4 months group showed an improvement of two or more diabetic retinopathy severity steps, compared to 15% in the sham group. The proportion of eyes developing PDR or CI-DME was reduced by 72–76% in the aflibercept arms, compared to the sham arm. The results of this study suggest a shift in the treatment paradigm for severe non-proliferative DR, providing support for initiating anti-VEGF therapy prior to the development of retinal neovascularization or CI-DME. The rationale for the paradigm shift must be carefully examined however, as 59% of eyes in the sham arm did not develop PDR or center-involved DME during the first year; so overall an anticipated 3 eyes need to be treated to benefit 1 eye. Furthermore, this study does not provide evidence at this time, that earlier treatment provides better long-term functional outcomes.

Dr. Jeffrey Heier presented 1-year results of the ProCon study which investigated the effectiveness of quarterly aflibercept in preventing conversion of high-risk eyes dry AMD to nAMD [14]. High risk was defined as intermediate dry AMD in one eye and a history of nAMD in the fellow eye. The study found that that rate of neovascular conversion was low at 1 year and that quarterly aflibercept did not reduce the rate of neovascular conversion, which was 9.38% in the active treatment arm and 6.35% in the sham arm. Conversion rates were higher if the fellow eye had neovascular disease for 2 years or less or if non-exudative choroid neovascularization was detected by optical coherence angiography (OCT-A) in the study eye. In high-risk eyes with quiescent choroidal neovascularization on OCT-A, the conversion rate was approximately 30% in both arms. The current management consensus is that anti-VEGF treatment be initiated after exudation develops.

## Geographic atrophy

There are no approved pharmacologic treatments for the advanced form of dry AMD: geographic atrophy (GA). GA is also commonly associated with nAMD, being observed in over 90% of nAMD eyes treated with anti-VEGF agents for 7 years [5]. The most promising future treatment approaches involve inhibition of specific components of the complement cascade, an inflammatory pathway believed to be a key driver of AMD pathogenesis [15].

Dr. Cedric Francois reported the latest results of APL-2, an inhibitor of C3 cleavage, for the treatment of GA (Apellis, Crestwood, KY). At the present time, APL-2 appears to be the most promising agent for treatment of GA. One hypothesis is that iC3b and C3d overloading of retinal pigment epithelial (RPE) cells causes an energy deficit leading to RPE cellular dysfunction and photoreceptor injury and death. In the completed phase 2 Filly trial, one-year results using monthly and every other month intravitreal delivery of APL-2 showed reductions in the area of GA growth of 20% and 29% respectively compared to sham injections [16]. The majority of the treatment effect was seen in the second 6 months. Treatment effect decreased after cessation of APL-2 treatment at 12 months, but the benefits achieved through 12 months were maintained to 18 months. Investigator-determined conversion to nAMD occurred in 9% and 21% of eyes in the every other month and monthly groups respectively; with prompt anti-VEGF treatment, visual acuity (VA) did not appear to be negatively affected. A phase III trial program is underway [17].

Of note, photobiomodulation, a non-pharmacologic therapy is also being investigated as a treatment strategy for dry AMD [18].

Dr. Jay Duker reported results from the Phase I HMR59 gene therapy trial involving 17 eyes (Hemera Biosciences), in which soluble CD59 is produced, which blocks the terminal stage of the complement pathway at the membrane attack complex (MAC) [19]. HMR59 is administered by an intravitreal injection. One-year results were impressive, showing an estimated 25% reduction of growth at the highest dose tested. Additional human clinical trials are planned.

## Drug delivery and devices

Drug delivery and device development are active spaces of innovation within retina.

Dr. Thomas Albini reported the 24-week results of the phase III Peachtree study which investigated the use of a unique suprachoroidal delivery system for treating uveitis related cystoid macular edema (CME) with triamcinolone acetonide (Clearside Biomedical) [20]. Eyes with CME secondary to all types of non-infectious uveitis were included. Eyes in the active treatment arm received suprachoroidal triamcinolone acetonide on day 1 and at 12 weeks. At 24 weeks, the primary treatment endpoint of best corrected VA gain of 15 or greater letters was achieved in 47% of the treatment group compared to 16% of the control group. CST improvements were 153 and 16 microns in the treatment and control groups respectively. There was no substantial signal of intraocular pressure increase or cataract progression following suprachoroidal steroid delivery compared to what may be anticipated with intravitreal steroid delivery, although this was a relatively short trial. Intraocular pressure adverse events were numerically higher in the control group (15.6%) compared to the treatment group (11.5%). New or worsening cataract complications occurred at comparable rates in both arms (approximately 7%). This delivery system seems promising for delivery of steroids to the posterior segment for CME secondary to uveitis.

Dr. Michael Ip speaking on behalf of the SCORE2 study group reported on the use of bevacizumab stored in 2 mL glass vials Pre-packaged bevacizumab in single-dose, pre-filled plastic syringes for intravitreal injection is widely available, but the shelf life is typically limited [21]. As part of the SCORE2 trial investigating the treatment of retinal venous occlusive disease, 2 mL glass vials of bevacizumab were used instead of prefilled plastic syringes. Studies showed that the bevacizumab stored in glass vials remained sterile at 21 months with intact anti-VEGF biological activity. This study suggests it may be valuable to prepare bevacizumab for intravitreal injection in glass syringes.

## Imaging retinal and choroidal vascular flow

OCT-A is emerging as a powerful research tool for investigating the pathogenesis of macular diseases. It has not yet found a role in routine clinical decision-making but some important advances were reported.

Dr. Harry Flynn described a study comparing wide field fluorescein angiography (FA) to wide field OCT-A. It is now clear that optimal imaging of diabetic eyes with suspected retinal neovascularization is improved with wide field imaging. The conventional 7-field approach using a 30-degree fundus camera captures significantly less retinal surface area compared to widefield imaging. Both FA and OCT-A demonstrate capillary non perfusion and both can image retinal neovascularization; only FA can demonstrate vascular leakage. OCT is a more versatile tool as in addition to providing angiographic data, at the same time, OCT structural data can be obtained and be precisely registered to the data obtained analyzing vascular flow patterns [22]. In addition, the capability of en-face sectioning with OCT permits cross sectional viewing of vitreous membranes. Widefield OCT and widefield OCT-A remain in the early stages of development, but this paper demonstrated that they may prove to be powerful tools moving forward.

Dr. Philip Rosenfeld presented a paper which discussed swept source OCT-A for studying the choriocapillaris (CC) in non-exudative AMD [23, 24]. OCT-A has emerged as a powerful tool for studying the choriocapillaris, providing a unique data set previously unavailable. On average, normal eyes demonstrate increasing choriocapillaris defects with age, particularly in the central macula. In AMD eyes with GA, growth rates of GA correlate more strongly with CC flow deficits away from the edge of GA. The role of CC flow deficits in GA pathogenesis is unknown and their clinical utility are being explored.

## Conclusions

In the early years of the twenty-first century, pharmaceuticals that block all VEGF isoforms transformed the management of nAMD. Fortunately, the wide-spread adoption of repeated intravitreal injections for management of exudative retinal diseases included nAMD, retinal venous occlusive diseases and DR brought remarkable benefit to innumerable people around the world. Nevertheless, much progress has yet to be made in the pursuit of optimizing long-term visual outcomes while minimizing treatment burdens for patients with retinal diseases.

The 2019 Angiogenesis, Exudation and Degeneration Symposium highlighted some of the most promising innovations anticipated to shape the management of retinal diseases. Novel pharmaceutical agents including brolucizumab and sunitinib, hardware solutions such as the PDS, and gene therapy approaches are being pursued in hopes of a better, more durable anti-VEGF effect. Randomized trials such as PANORAMA and ProCon are investigating the role of earlier pharmacologic intervention in high-risk eyes with DR and dry AMD respectively. The complement cascade is being directly targeted in hopes of finding a first-in-class therapeutic to slow the expansion of GA secondary to AMD. The suprachoroidal space is being investigated as a route for drug delivery. Finally, improved hardware and software algorithms continue to advance OCT-based imaging, affording a more complete understanding of normal and pathologic retinal and choroidal processes.

## Data Availability

Not applicable.
